# Protective and pathologic immune responses in human tegumentary leishmaniasis

**DOI:** 10.3389/fimmu.2012.00301

**Published:** 2012-10-04

**Authors:** Lucas P. Carvalho, Sara Passos, Albert Schriefer, Edgar M. Carvalho

**Affiliations:** ^1^Serviço de Imunologia, Complexo Hospitalar Universitário Professor Edgard Santos, Universidade Federal da BahiaSalvador, Bahia, Brazil; ^2^Instituto de Ciências da Saúde, Universidade Federal da BahiaSalvador, Bahia, Brazil; ^3^Instituto Nacional de Ciência e Tecnologia em Doenças Tropicais (Conselho Nacional de Desenvolvimento Científico e Tecnológico/Ministério da Ciência e Tecnologia)Salvador, Bahia, Brazil

**Keywords:** Leishmaniasis, immune response, *Leishmania braziliensis*

## Abstract

Studies in the recent years have advanced the knowledge of how host and parasite factors contribute to the pathogenesis of human tegumentary leishmaniasis. Polymorphism within populations of *Leishmania* from the same species has been documented; indicating that infection with different strains may lead to distinct clinical pictures and can also interfere in the response to treatment. Moreover, detection of parasite genetic tags for the precise identification of strains will improve diagnostics and therapy against leishmaniasis. On the host side, while a predominant Th1 type immune response is important to control parasite growth, it does not eradicate *Leishmania* and, in some cases, does not prevent parasite dissemination. Evidence has accumulated showing the participation of CD4^+^ and CD8^+^ T cells, as well as macrophages, in the pathology associated with *L. braziliensis*, *L. guayanensis*, and *L. major* infection. The discovery that a large percentage of individuals that are infected with *Leishmania* do not develop disease will help to understand how the host controls *Leishmania* infection. As these individuals have a weaker type 1 immune response than patients with cutaneous leishmaniasis, it is possible that control of parasite replication in these individuals is dependent, predominantly, on innate immunity, and studies addressing the ability of neutrophils, macrophages, and NK cells to kill *Leishmania* should be emphasized.

## INTRODUCTION

Tegumentary leishmaniasis (TL), caused by protozoan parasites of the genus *Leishmania*, is a major health problem in many regions of the world. After been transmitted by sand flies, *Leishmania* parasites infect human macrophages and dendritic cells (DCs), causing a wide spectrum of clinical manifestations, including self-healing skin lesions, cutaneous leishmaniasis (CL), disseminated leishmaniasis (DL), mucosal leishmaniasis (ML), and diffuse cutaneous leishmaniasis (DCL). Around 10% of individuals living in *L. braziliensis* transmission areas have evidence of exposure to *Leishmania*, as determined by a positive *Leishmania* skin test (LST), but do not develop disease (sub-clinical infection). Host, parasite, and vector factors participate in the pathogenesis of leishmaniasis. More than 20 species of *Leishmania* cause human disease and CL is the most common clinical picture of TL. In the Old World the most important species are *L. major*, *L. tropica*, and *L. aethiopica*, and in the New World, *L. braziliensis*, *L. amazonensis*,* L. mexicana*, *L. guyanensis*, and *L. panamensis*. This review will address the role of parasites, and innate and adaptive immunological responses in the pathogenesis of TL.

## PARASITE FACTORS IN TEGUMENTARY LEISHMANIASIS

Productive infections with *Leishmania *spp result in visceral or several tegumentary disorders (Murray et al., 2005). This reflects the substantial variability among the etiological agents at the subgenus level with many species described as human pathogens, which may be subdivided into the *Leishmania Leishmania* subgenus that comprises the *L. tropica*, *L. donovani*, and *L. mexicana* complexes of species, and the subgenus *Leishmania Viannia* that consists in the *L. braziliensis* complex of species (Thomaz-Soccol et al., 1993a,b; Shaw, 1994; Croan et al., 1997). Many, if not all, of these species present a high degree of intra-species genetic and phenotypic polymorphism, which is accompanied by a spectrum of clinical presentations in the infected human host. For example, an ample variability has been reported for *L. braziliensis* (Kahl et al., 1991; Gomes et al., 1995; Saravia et al., 1998, 2002; Ishikawa et al., 2002; Cupolillo et al., 2003; Schriefer et al., 2004), which causes at least three well documented forms of TL: CL, ML, and the emerging DL (Costa et al., 1986; Carvalho et al., 1994b; Azulay and Azulay Júnior, 1995; Turetz et al., 2002; Guerra et al., 2011).

Although a thorough understanding of the mediators of infection outcome is lacking, some mechanisms have been unveiled. Parasite persistence due to evasion of immunity is one factor influencing disease duration and clinical outcome toward more severe forms of leishmaniasis, in part through arginine metabolism. Depending on the type of arginase that the host cell expresses, arginine metabolism may result in the production of nitric oxide (NO), or in L-ornithine. NO is toxic for the parasite, while L-ornithine is essential for *Leishmania* growth (Iniesta et al., 2001). Besides, the parasite’s own arginase was shown to influence infectivity. *L. mexicana* knocked out for arginase activity led to significantly attenuated infection of mice and had poorer survival inside macrophages than the wild type strain (Gaur et al., 2007). This seems in concordance with reports which show that the insulin-like growth factor one promotes *in vivo* and *in vitro* growth of different species of *Leishmania*, at least in part, by activating arginase (Vendrame et al., 2007). A possible clinical expression of these findings may be the observation that promastigotes of *L. amazonensis* and *L. braziliensis* that are resistant to NO *in vitro* are associated with poorer outcomes of the patients they were isolated from (Giudice et al., 2007).

**FIGURE 1:**
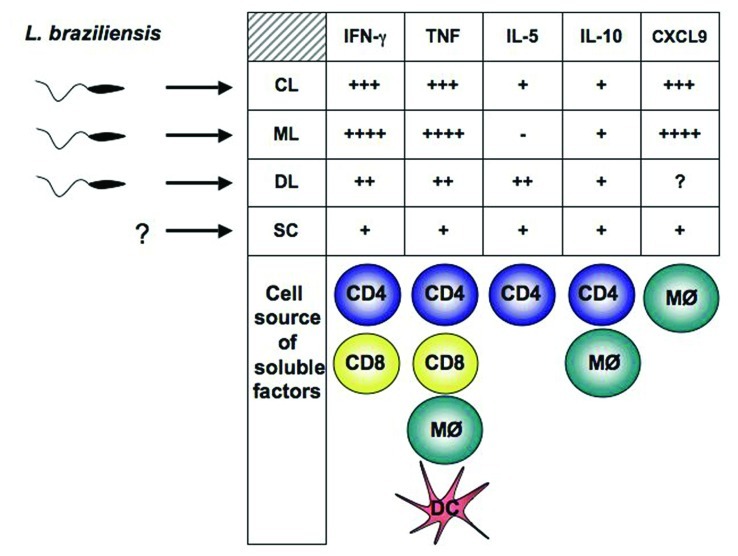
** Parasite and host factors contribute to disease outcome in human *L. braziliensis* infection.**Parasite polymorphisms are associated with clinical forms of the disease. The type of immune response developed by the host, determine whether parasite growth will be controlled or dissemination will occur. Detection of *Leishmania* in blood from SC individuals has not been achieved. Exaggerated pro-inflammatory immune response leads to tissue damage and ulcer development. Cells known to contribute to cytokines and CXCL9 production in *L. braziliensis*-infected patients are represented below the corresponding soluble factor. CL, cutaneous leishmaniasis; ML, mucosal leishmaniasis; DL, disseminated leishmaniasis; SC, sub-clinical.

Different reports indicate that parasite resistance to hydrogen peroxide may also play a role in more severe forms of leishmaniasis. Clones of *L. guyanensis* capable of metastasization in golden hamsters present cytoplasmic peroxiredoxin and peroxidase activities different from those of non-metastatic parasites (Acestor et al., 2006), while laboratory strains of *L. guyanensis* with metastatic phenotype present isoforms of tryparedoxin peroxidase and elongation factor-1 beta different from those of non-metastatic strains (Walker et al., 2006). Two reports described an increased frequency of mucosal involvement among human cases caused by certain *L. braziliensis* strains in Colombia (Saravia et al., 1998, 2002). Length of cutaneous disease in those infected with parasites of a “mucosal-prone” *L. braziliensis* zymodeme was also significantly longer than that caused by other strains (Saravia et al., 2002). Another study performed in one of the regions with greatest endemicity for ATL in Brazil described a complex population of *L. braziliensis* made up of several different clones of the parasite affecting leishmaniasis patients and detected a statistically significant association between parasite genotype and clinical outcomes toward CL, ML, or DL (**Figure 1**; Schriefer et al., 2004). Interestingly, a follow-up study in the same region identified that the distribution of ML and DL, which are more aggressive forms of TL, differed significantly across the affected area and that geographic distribution of TL forms also seemed to be influenced by the strain of *L. braziliensis* (Schriefer et al., 2009). Nevertheless, the most compelling putative mechanism leading to ML involves the infection of *L. guyanensis* strains with the *Leishmania* RNA virus-1 (LRV-1; Ives et al., 2011). In experimental mice, the metastasizing *L. guyanensis* parasites presented higher LRV-1 burden than non-metastasizing strains. It was found that the increased LRV-1 burden stimulated the host Toll-like receptor 3 (TLR3) and induced pro-inflammatory cytokine and chemokine production by the macrophages. If this mechanism overlaps what occurs in human disease pathology, it would ultimately result in the strong inflammatory response and tissue destruction observed in the ML patients.

## INNATE IMMUNE RESPONSE IN HUMAN TEGUMENTARY LEISHMANIASIS

The development of a protective immune response to intracellular pathogens requires the coordinated action of cells from innate and adaptive immunity. After *Leishmania* infection, different cell types of the innate immune response can interact with the parasite. Although macrophages are the major host cells for *Leishmania*, reports using animal models have shown that neutrophils and DCs are also able to uptake *Leishmania* parasites early after infection. Neutrophils provide an important link between innate and adaptive immunity during parasitic infections. These cells can interact with monocytes, DCs, and T and B lymphocytes through cell–cell contact or secreted products, driving inflammatory responses involved in host defense and tissue repair (Nathan, 2006; Charmoy et al., 2010). Cooperation between neutrophils and macrophages contributes to both resistance and susceptibility to *Leishmania* infection in experimental models. Thus, neutrophils from BALB/c mice induce intramacrophagic killing of *L. braziliensis*, which was dependent on TNF and reactive oxygen species (Novais et al., 2009). Also, phagocytosis and elimination of *Leishmania* amastigotes by neutrophils was shown in the later stages of the disease (Daboul, 2010). Neutrophils have a short life span and are constitutively programmed to die by apoptosis. Clearance of apoptotic cells by macrophages is associated with anti-inflammatory mediators such as TGF-beta and PGE2, which inhibit macrophage activation (Voll et al., 1997; Fadok et al., 1998), whereas, phagocytosis of necrotic neutrophils induces macrophage activation through production of pro-inflammatory mediators (Savill, 2000). The effect of human necrotic and apoptotic neutrophils in response to *L. amazonensis* infection was investigated. It was observed that apoptotic, but not viable, neutrophils increased the parasite burden through a mechanism dependent on TGF-beta and PGE2. In an opposite way, interaction of necrotic neutrophils with *L. amazonensis*-infected macrophages decreased the infection rate as well as the parasite burden (Afonso et al., 2008).

Classically activated macrophages secrete IL-12, produce oxygen reactive species and are the main cells involved in intracellular parasite killing, whereas alternatively activated and regulatory macrophages are permissive to parasites growth (Mosser and Zhang, 2008; Wanasen and Soong, 2008; Martinez et al., 2009). Although classically activated macrophages have leishmanicidal machinery, some species of *Leishmania* parasites have developed ways to escape immune events (Mosser and Zhang, 2008). For instance, studies have shown that *L. braziliensis* infection of macrophages lead to proteasome-mediated degradation of STAT-1, production of regulatory cytokine, TGF-beta, and impairment in adhesion to extracellular matrix (Barral et al., 1993; Forget et al., 2005; Pinheiro et al., 2006). However, these *in vitro* studies are quite contradictory with the *in vivo* scenario of *L. braziliensis*-infected patients, where exaggerated inflammatory response is observed and low amounts of parasites are detected. Differences in macrophage responses to *Leishmania* parasites have been documented. For example, macrophages from individuals with sub-clinical infection control parasite growth more efficiently than macrophages from CL patients as observed in human *L. braziliensis* infection (Bosque et al., 1998; Giudice et al., 2012), pro-inflammatory chemokines, such as CCL2, CXCL-9, and CXCL-10, secreted predominantly by macrophages, are higher in CL and ML than in individuals with SC *L. braziliensis* infection. As these molecules activate and recruit macrophages and T cells to the lesion site, they may participate in the pathology.

NK cells represent one of the first lines of defense in the immune reaction after invasion of *Leishmania* parasites. Those cells can produce IFN-γ that will activate macrophages to kill *Leishmania*. Absence or low number of NK cells, and impairment of NK cells response were documented in lesions from patients with CL. The possible explanation for this result, can be the fact that direct contact points found between *Leishmania *promastigotes and naïve human NK cells causes immediate destruction of NK cells in a non-apoptotic way (Lieke et al., 2011). In this context, a role for gp63 in inhibition of human NK cells proliferation has been demonstrated (Lieke et al., 2011). Indirect evidence of the role of NK cells to the protective immune response against *Leishmania* come from the observation that, in individuals with SC infection as well as individuals without exposure to *Leishmania*, NK cells is the main source of IFN-γ upon stimulation *in vitro* with *L. aethiopica* (Maasho et al., 1998; Nylen et al., 2003).

In leishmaniasis a large variety of cells contribute to the production of IL-10, including DCs and macrophages. While IL-10 plays an important role in down regulating Th1 immune response, decreasing lymphocyte proliferation and production of IFN-γ in patients with visceral leishmaniasis (Carvalho et al., 1994a; Nylen and Sacks, 2007), the role of IL-10 in the pathogenesis of TL is not so clear. IL-10 facilitates parasite growth and it may play an important role in the initial phase of *Leishmania* infection, contributing to the establishment of the parasite. As most of the studies evaluating the role of IL-10 in leishmaniasis are related to its indirect effects in T cells function, this subject will be discussed later in the section of adaptative immune response.

Dendritic cells are essential for an effective immune response against most pathogens. Prior to infection, DCs survey the tissues and the lymphoid organs as “immature” cells. *Leishmania* killing process, require that DCs uptake *Leishmania* and *Leishmania* antigens, migrate to lymph nodes, present antigen and prime T cells to produce IFN-γ. For efficiently present antigen, DC has to undergo through a maturation program, which includes up-regulation of molecules such as MHC II, CD80, and CD86 and production of pro-inflammatory cytokines such as IL-12 and TNF. In order to avoid activation of cells from adaptative immune response, *Leishmania* have developed mechanisms to inhibit DC function. Many works have been performed using mouse DCs and conflicting results have been published regarding ability of *Leishmania* to inhibit these cells functions. While some studies documented mononuclear phagocytes activation upon *Leishmania* infection, others did not see change on activations markers (De Trez et al., 2004; Sanabria et al., 2008). *In vitro* single cell-based analysis have revealed that while *Leishmania*-infected DCs remains immature, bystander ones up-regulate MHC II and costimulatory molecules (Carvalho et al., 2008). Investigations using human cells have documented that certain primary human DC subsets obtained *ex vivo* uptake *Leishmania* and release IL-12. This is in contrast to prior studies indicating that human DC do not contribute to primary immunity against *Leishmania* (Marovich et al., 2000; McDowell et al., 2002; Favali et al., 2007; Revest et al., 2008; Griewank et al., 2010). The discrepancy between these studies may have to do with different parasite species and life-cycle stages, as well as different sources of cells used.

## CELL-MEDIATED IMMUNE RESPONSE IN HUMAN TEGUMENTARY LEISHMANIASIS

Since the early studies in cell-mediated immune response to intracellular pathogens in the beginning of the 80s, much progress has been made for the understanding of how host fight *Leishmania* pathogens. Works using mouse models, intent to elucidate immune events important for *Leishmania* killing, have revealed that reactive oxygen species produced by IFN-γ-activated mononuclear phagocytes, is the main mechanism used by infected macrophages to destroy *Leishmania*. Thus, while in BALB/c mice infected with *L. major* Th2 immune response prevails allowing parasites to multiplicate, C57BL/6 mice are able to control infection with strong Th1 immune response. In humans, the Th1/Th2 paradigm does not quite explain the natural outcome of the disease. TL patients may present different clinical forms of diseases with distinct pattern of immune response. Clinically, patients may develop single or multiple cutaneous ulcers, nodular, papular, or acneiform lesions, and although Th1 responses be the desired for parasite killing, number of lesions is not associated with the type of immune response developed after infection. For example, patients infected with *L. amazonensis* may develop DCL, a disease characterized by the presence of many nodular lesions. Lymphocytes from DCL patients do not produce IFN-γ upon *in vitro* stimuli with *Leishmania* antigen, which is associated with parasite proliferation and dissemination along the body of these individuals. Differently, individuals infected with *L. braziliensis*, may develop an emerging form of leishmaniasis, also with multiple lesions denominated DL. In these individuals high production of TNF and IFN-γ is documented, both in blood and tissue, and they develop ulcerative lesions similar to that observed in CL and ML (**Figure 1**; Machado et al., 2011). Therefore, while in the absence of Th1 immune response, as observed in DCL, parasite multiply and disseminate, an exaggerated type 1 immune response is observed in patients with DL, CL, and ML. However, as *Leishmania* is not eradicated the tissue damage in these forms of leishmaniasis is associated with the inflammatory reaction.

It is quite difficult to evaluate human immune response early after *Leishmania* infection. Studies in an endemic area of *L. braziliensis* transmission have documented that before cutaneous ulcer develops, lymphadenopathy is observed in most cases (Barral et al., 1995). In a few weeks, a papular or exulcerative lesion appears. Biopsy of these initial lesions showed that parasites are present, although in very low numbers, and mononuclear cells start to infiltrate as angiogenesis occurs. During this phase IFN-γ levels are low, but significant amount of TNF can be observed in *Leishmania* antigen-stimulated peripheral blood mononuclear cells (PBMCs) supernatants (Rocha et al., 1999; Unger et al., 2009). Inflammatory infiltrate composed, predominantly, by mononuclear phagocytes, T and B lymphocytes and plasma cells, is already documented and increases as ulceration develops. The low production of pro-inflammatory cytokines in the early phase of CL is due, in part, to presence of IL-10, as neutralization of this cytokine enhances IFN-γ production (Rocha et al., 1999). Usually, 1–2 weeks after the appearance of the papule and exulceration, a classic CL ulcer is observed. Development of the classical ulcer coincides with a great increase in TNF and IFN-γ and low levels of IL-10 (Bacellar et al., 2002; Unger et al., 2009). High amounts of IFN-γ and TNF are observed in both, *Leishmania* antigen-stimulated PBMC cultures, and in the ulcer of CL patients. The levels of inflammatory cytokines in *Leishmania* antigen-stimulated PBMC cultures from patients with ML, is even higher than those documented in CL (Bacellar et al., 2002; Carvalho et al., 2007). This is also true at lesion site (Faria et al., 2005). Evidences for the contribution of TNF for immunopathology in TL, has been accumulated: (1) there is a positive correlation between lesion size and TNF levels in PBMC cultures stimulated with *Leishmania* antigen (Antonelli et al., 2005); (2) TNF levels fall after therapy of CL and ML (Da-Cruz et al., 2002); (3) Patients treated with pentavalent antimonial combined with pentoxifylline, a TNF inhibitor, have a better rate of cure than those treated with pentavalent antimonial alone (Lessa et al., 2001; Machado et al., 2002); (4) Pentoxifylline combined with antimony therapy cure CL and ML patients refractory to antimony therapy (Lessa et al., 2001; Bafica et al., 2003).

The enhanced IFN-γ and TNF production in CL and ML is not due to absence of IL-10. Although low amounts of IL-10 are found in supernatants from *Leishmania* antigen-stimulated PBMC from CL and ML individuals, studies in lesions of these patients have documented presence of IL-10-secreting macrophages and regulatory T cell (CD4^+^CD25^+^Foxp^3^^+^; Campanelli et al., 2006; Bourreau et al., 2009; Faria et al., 2009). The participation of regulatory T cells in the regulation of immune response in experimental leishmaniasis has been intensively investigated. IL-10 secreted by Tregs are involved in persistence of parasites, regulation of Th2 cell expansion, and control of cell-mediated lesion development in leishmaniasis (Powrie et al., 1994; Sacks and Kamhawi, 2001; Aseffa et al., 2002; Xu et al., 2003; Mendez et al., 2004; Ji et al., 2005). Regulatory T cells isolated from lesion from CL patients infected with *L. braziliensis* and *L. guyanensis* were able to inhibit proliferation of T cells to polyclonal and *Leishmania* antigen stimuli (Campanelli et al., 2006, 2010; Bourreau et al., 2009). In human, an obvious question to be addressed has to do with the discrepancy between presence of IL-10 in tissue and lack of regulation of inflammatory response in ML. One explanation would be that cells arriving at lesion site have already effector characteristics, and can no longer be modulated by the effects of IL-10. In ML, lack of response to IL-10 can be in part explained by the down-regulation of IL-10 receptor, as lesions from ML patients have a decrease in the number of cells expressing IL-10 receptor and a decrease in the intensity of expression of this receptor when compared with CL patients (Faria et al., 2005). However, studies have to be performed for the better understanding of the dynamic of IL-10 receptor expression in uninfected controls as well as in leishmaniasis patients in response to *Leishmania* antigens. IL-27 is a regulatory cytokine of interest in infectious disease. Although initial studies have identified IL-27 as an inflammatory cytokine since it can promote Th1 responses by enhancing T-bet expression in CD4^+^ T cells, later reports have shown that IL-27 can down-regulate T cell activity both, dependent and independent of IL-10 demonstrated in human visceral leishmaniasis (Hunter et al., 1994; Ansari et al., 2011). Expression of IL-10 and IL-27 were similar in CL patients and in individuals with sub-clinical *L. braziliensis* infection (Novoa et al., 2011).

The attempt to down-regulate *Leishmania* antigen-induced IFN-γ production in ML using *in vitro* monoclonal antibody to IL-12 or IL-15, failed, suggesting that patients with ML have a portion of T cell population fully differentiated that no longer depends on these cytokines to differentiate/survive and induce inflammation. In fact, the *ex vivo* analysis of peripheral blood reveled that ML patients have increased population of CD4^+^ T cells expressing CD69, CD25, and CD62L^dim^, when compared to CL individuals (Gaze et al., 2006; Carvalho et al., 2007). It is well known that the magnitude of inflammatory response can be influenced by host genetic. For example, familial aggregation in ML has been documented and polymorphisms of genes encoding inflammatory proteins (cytokines and chemokines) are associated with CL and ML (Castellucci et al., 2011). As mentioned before, parasite genetic factors also interfere on inflammatory responses, as *Leishmania* antigens isolated from CL patients induces more TNF and IFN-γ production than those from DL patients (Castellucci et al., 2006; Leopoldo et al., 2006).

CD8^+^ T cells are predominantly recognized by its cytotoxic characteristics, but they can also have regulatory properties. In mice, CD8^+^ T cell play an important role in the protection against *Leishmania* infection (Wang et al., 1993). In lesions of CL patients infected with *L. guayanensis*, CD8^+^ T cells expressing IL-10 are documented (Bourreau et al., 2009). Thus, it is possible that although IL-10 is not able to control the exaggerated inflammatory response, it does facilitate the maintenance of parasites in tissue. CD8^+^ T lymphocytes kill *L. braziliensis*-infected cells *in vitro*, and killing was greater in ML patients than in CL patients (Brodskyn et al., 1997). However, as *Leishmania* persists in CL and ML patients despite the presence of CD8^+^ T cells and these cells also produce pro-inflammatory cytokines, it is possible that they also participate in the pathology, by killing epithelial cells that expresses *Leishmania* antigen. In favor of this hypothesis, it has been shown that the frequency of CD8^+^ T cells expressing granzyme in tissue of CL patients is higher when compared to patients in the early phase of CL, and that the frequency of CD8^+^ T cells expressing granzyme, is directly associated with the intensity of the inflammatory reaction in CL ulcers (Faria et al., 2009).

Several studies have established a role for IL-17 in susceptibility or protection to intracellular parasites infections. In a susceptible mouse model for *L. major*, absence of IL-17 resulted in smaller lesions (Lopez Kostka et al., 2009). Differently, in *L. braziliensis* mouse model of leishmaniasis, self-healing lesions were associated with presence of IL-17 (Vargas-Inchaustegui et al., 2008). In humans, IL-17 has been found in lesions of CL and ML patients and PBMC from these individuals produce this cytokine in response to *Leishmania* antigen (Bacellar et al., 2009; Boaventura et al., 2010). However, IL-17 was not associated with pathology as determined by lesion size or presence of mucosal disease (Bacellar et al., 2009). In other infectious diseases, the main function of IL-17 is to, indirectly, recruit neutrophils to inflammatory site (Charmoy et al., 2007). However, in spite of the presence of IL-17 in lesion of *L. braziliensis*-infected individuals, the majority of the studies have documented mononuclear cells infiltrate in lesion of CL and ML. Importantly, a recent report showed neutrophils in lesion of ML patients, suggesting that these cells may play a role in tissue damage in ML (Boaventura et al., 2010). Different from patients with CL and ML, individuals with sub-clinical *L. braziliensis* infection produce low amounts of IFN-γ and TNF (Follador et al., 2002), are able to kill *Leishmania* and do not develop disease. The low production of pro-inflammatory cytokines in SC infection is not due to regulatory mechanisms mediated by IL-10 and IL-27 (Novoa et al., 2011). In contrast with the low production of TNF, these individuals produce IL-17 levels similar to the ones observed in CL and ML patients (Novoa et al., 2011). As IL-17 has been associated with protection in VL (Pitta et al., 2009). It is important to determine if this cytokine play a role in the control of TL.

## PARASITE AND HOST FACTORS IN THERAPY FOR TEGUMENTARY LEISHMANIASIS

There are a large variety of therapies for TL, that includes local treatments as cryotherapy, thermotherapy and local antigens application, and systemic therapies, being the more common pentavalent antimonials, pentamidine and liposomal amphotericin B. While the efficacy of local therapies was documented in infections with *L. major*, *L. tropica*, and *L. mexicana, *pentamidine is the drug of choice for *L. guyanensis*, and pentavalent antimonials for *L. braziliensis* infection. It has been well documented that patient response to antimonials varies according to the species of the parasite they are infected with, and even responses to drugs to parasite species belonging to the same complex can be heterogeneous (Arevalo et al., 2007). It has been shown that sensitivity of *L. braziliensis* promastigotes to antimony is greater than that of other species of the same complex (Azeredo-Coutinho et al., 2007). However, to underscore the complexity of this issue the infection with *L. braziliensis* and *L. peruviana* was found to be a risk factor for antimony failure in Peru (Llanos-Cuentas et al., 2008), and different reports show that the efficacy of these drugs vary even within a single species like *L. braziliensis *(Saldanha et al., 1999; Andersen et al., 2005).

A growing literature reveals that *Leishmania* spp. are able to develop resistance to antimony, which is abroad used drug to treat leishmaniasis patients. The occurrence of resistant strains and of cases failing treatment due to parasite variants has been reported in highly affected countries, like Iran (Hadighi et al., 2006), Peru (Llanos-Cuentas et al., 2008), and Brazil (Arevalo et al., 2007; Azeredo-Coutinho et al., 2007), involving species as diverse as *L. tropica*, *L. donovani*, and *L. braziliensis* (Hadighi et al., 2006; Arevalo et al., 2007; Azeredo-Coutinho et al., 2007; Samant et al., 2007; Llanos-Cuentas et al., 2008). The phylogenetic distance among these species suggest that these parasites must be capable of adapting for drug pressure by diverse mechanisms.

Although detailed description of drug resistance mechanisms is still scarce for this genus, the detection of parasite markers for drug resistance/susceptibility phenotypes seems to be an attainable goal. In one such case, it has been shown that a group of glycoproteins collectively called proteophosphoglycans (PPGs) is overexpressed on the surfaces of promastigotes and amastigotes of stibogluconate-resistant field isolates of *L. donovani* from India (Das Gupta et al., 1991; Samant et al., 2007). Although the role PPGs may play in the mechanism of resistance is unknown, the authors pointed out that their overexpression could be explored as a phenotypic marker of likely drug resistance among parasites isolated with diagnostic purposes.

In addition to parasites factors, host immunologic factors are also important for therapy in TL. Patients with DCL, the anergic form of TL, have impairment in the Th1 type immune response to *Leishmania* antigens and failed to all forms of therapy or relapse after apparent successful therapy. In one report, the use of recombinant IFN-γ associated with antimony induced cure in VL patients (Badaro et al., 1990). Thus, this may also consist in a candidate therapy for better managing DCL. A better understanding of the immunologic mechanisms of tissue damage in leishmaniasis may also help to improve the management of leishmaniasis. Granulocyte colony stimulate factor (GM-CSF) improves antigen presentation and also down modulate the exaggerated inflammatory response in CL. Topical or systemic GM-CSF associated with antimonial therapy is more effective than antimony alone and cure CL patients refractory to antimonial therapy (Almeida et al., 2005). The documentation that TNF plays a pivotal role in tissue damage in *Leishmania* infection, have led to use of pentoxifylline as adjuvant therapy in CL and ML. Pentoxifylline down-modulates TNF as well as pro-inflammatory chemokines, and when associated with antimony therapy is more effective than antimony alone, accelerating healing time of CL and ML and curing CL and ML patients refractory to antimony therapy (Lessa et al., 2001; Bafica et al., 2003; Machado et al., 2007).

## Conflict of Interest Statement

The authors declare that the research was conducted in the absence of any commercial or financial relationships that could be construed as a potential conflict of interest.
